# The effectiveness of glucocorticoid treatment in post-COVID-19 pulmonary involvement

**DOI:** 10.1186/s41479-023-00123-7

**Published:** 2024-02-05

**Authors:** Jan Mizera, Samuel Genzor, Milan Sova, Ladislav Stanke, Radim Burget, Petr Jakubec, Martin Vykopal, Pavol Pobeha, Jana Zapletalová

**Affiliations:** 1 Department of Respiratory Medicine and Tuberculosis, Faculty of Medicine and Dentistry Palacky University Olomouc, University Hospital Olomouc, Olomouc, Czech Republic; 2 Center for Digital Health, Faculty of Medicine and Dentistry, Palacky University Olomouc, Hnevotinska 976/3, Olomouc, 779 00 Czech Republic; 3 Department of Respiratory Medicine and Tuberculosis, Faculty of Medicine and Dentistry Masaryk, University Hospital Brno, University Brno, Brno, Czech Republic; 4 Dept. of Telecommunications, Faculty of Electrical Engineering and Communication, Brno University of Technology, Brno, Czech Republic; 5 Department of Respiratory Medicine and Tuberculosis, L.Pasteur University Hospital and Faculty of Medicine P.J. Safarik University Kosice, Kosice, Slovakia; 6 Department of Medical Biophysics, Faculty of Medicine and Dentistry Palacky University Olomouc, Olomouc, Czech Republic

**Keywords:** Post-covid syndrome, Pulmonary fibrosis, Corticosteroids, Watchful waiting, Pulmonary function

## Abstract

**Rationale:**

Persistent respiratory symptoms following Coronavirus Disease 2019 (COVID-19) are associated with residual radiological changes in lung parenchyma, with a risk of development into lung fibrosis, and with impaired pulmonary function. Previous studies hinted at the possible efficacy of corticosteroids (CS) in facilitating the resolution of post-COVID residual changes in the lungs, but the available data is limited.

**Aim:**

To evaluate the effects of CS treatment in post-COVID respiratory syndrome patients.

**Patients and methods:**

Post-COVID patients were recruited into a prospective single-center observational study and scheduled for an initial (V1) and follow-up visit (V2) at the Department of Respiratory Medicine and Tuberculosis, University Hospital Olomouc, comprising of pulmonary function testing, chest x-ray, and complex clinical examination. The decision to administer CS or maintain watchful waiting (WW) was in line with Czech national guidelines.

**Results:**

The study involved 2729 COVID-19 survivors (45.7% male; mean age: 54.6). From 2026 patients with complete V1 data, 131 patients were indicated for CS therapy. These patients showed significantly worse radiological and functional impairment at V1. Mean initial dose was 27.6 mg (SD ± 10,64), and the mean duration of CS therapy was 13.3 weeks (SD ± 10,06). Following therapy, significantly better improvement of static lung volumes and transfer factor for carbon monoxide (DLCO), and significantly better rates of good or complete radiological and subjective improvement were observed in the CS group compared to controls with available follow-up data (*n* = 894).

**Conclusion:**

Better improvement of pulmonary function, radiological findings and subjective symptoms were observed in patients CS compared to watchful waiting. Our findings suggest that glucocorticoid therapy could benefit selected patients with persistent dyspnea, significant radiological changes, and decreased DLCO.

## Introduction

Post-COVID-19 (Coronavirus Disease 2019) syndrome is a set of respiratory and non-respiratory symptoms that persist for more than 3 months after the infection with the Severe acute respiratory syndrome coronavirus 2 (SARS-CoV-2). These symptoms should have a pathophysiological connection with the COVID-19 and should not be explicable by another cause [1, 2]. The term measurable post-COVID disability can be used for patients presenting with abnormal findings on CXR, high-resolution computed tomography (HRCT), or pulmonary function testing, in the absence of subjective symptoms [3].

The reported prevalence of post-COVID lung fibrosis varies greatly across studies, ranging from 6% [3] to 44%, [4] likely due to methodological and terminological discrepancies, such as the lack of distinction between interstitial inflammation (reversible changes) and fibrosis (permanent changes). The most frequently described radiological and histological findings in patients with respiratory form of post-COVID-19 syndrome are consistent with organizing pneumonia (OP), followed by non-specific interstitial pneumonia (NSIP) [5]. Other radiological findings should raise suspicion of a different cause, or a pre-existing respiratory disease.

Multiple pathophysiological pathways and mechanisms are involved in the fibrotic remodeling of lung tissue, especially the downregulation of ACE-2 receptors after the binding of SARS-CoV-2 virions, resulting in increased pro-inflammatory and pro-fibrotic activity. This leads to the upregulation of various effector molecules (matrix metalloproteinases 1 and 7, interleukins: IL-1, 6 and 8, galectin-3 mediated pathways, transforming growth factor-β, TNF-α, ICAM, VCAM etc [6, 7]. Moreover, pulmonary vasculopathy and the resultant hypoxic remodeling may also be involved [8, 9]. In the majority of patients, the potentially fibrogenic inflammatory process seems to be self-limited and might resolve without any therapy in the post-acute stage of the COVID-19 [10, 11].

In the case of persistent lung interstitial involvement after COVID-19 pneumonia, systemic glucocorticoids (CS) are frequently used [5, 12]. The treatment with CS leads to downregulation of a broad spectrum of pro-inflammatory and pro-fibrotic molecules (IL-1, 2, 4, 5, 8, 13; ICAM, VCAM, etc.) and can thus help the resolution of inflammatory and fibrogenic changes through multiple pathways [13–19]. However, the CS seem to be most effective prior to the development of definitive fibrotic changes, therefore appropriate timing of the treatment is likely essential [5]. The existing literature suggests possible benefits of CS for selected individuals with respiratory form of post-COVID-19 syndrome, especially in cases of persistent organizing pneumonia, but their general use is currently not recommended [20–26]. The numerous and potentially serious systemic and organ specific side effects of CS are well described in the literature [27–29].

Several studies of the use of CS in post-COVID patients have been published to date, [5, 30, 31]  hinting at the possible efficacy of CS in the respiratory post-COVID-19 syndrome. These studies have brought important insights to the efficacy of CS, however, the cohort size in these studies was usually small, [5, 30] and most of the studies did not contain a control group [5, 31]. Additionally, some studies involved only limited [31] or no [30] pulmonary function tests (PFT). The present study aims to expand on the current knowledge by providing a larger, robust dataset, and more importantly, a control group with patients managed by watchful waiting (WW).

This study aims to answer two questions: Firstly, how do the patients treated by CS compare to the patients managed by WW with respect to the rate of improvement of parameters of pulmonary function, radiological findings and subjective symptoms? And secondly, what are the characteristics of the patients who are treated with CS in real-life clinical practice?

## Materials and methods

### Recruitment

From May 13, 2020, to January 11, 2022, more than three thousand patients were evaluated in the newly established post-COVID ambulatory center at the Department of Pulmonary Diseases and Tuberculosis, University Hospital Olomouc, Czech Republic. The availability of the post-COVID ambulatory was publicly announced on the website of the University Hospital Olomouc. The patients were invited to participate in a longitudinal observational study of COVID-19 survivors. Those who agreed to participate in the study signed an informed consent form. For the 12 adolescent patients the consent form was signed by a parent. Non-participants received the same level of medical care as study participants, but their clinical data were not included in the dataset.

The inclusion criteria for the study were: a minimum age of 15, and a previous COVID-19 infection, defined as: a history of a positive SARS-CoV-2 PCR test, a history of a positive SARS-CoV-2 antigen test coupled with typical symptoms, or positive levels of SARS-CoV-2 IgM antibodies in non-vaccinated individuals who experienced typical symptoms. No primary exclusion criteria were defined, however the patients with a probable refutation of SARS-CoV-2 infection were excluded post hoc (patients that had no evidence of having had covid i.e., an absence of a positive PCR or antigen test for SARS-CoV-2 and undetectable SARS-CoV-2 antibody levels in serum). Additionally, the patients with insufficient data (such as missing baseline pulmonary function testing), and the patients on chronic CS medication (rheumatoid arthritis, solid organ transplant recipients, etc.), or patients with newly initiated CS therapy for reasons other than post-COVID respiratory sequelae (bronchial asthma exacerbation, newly diagnosed sarcoidosis, etc.) were excluded post-hoc from the statistical analysis to avoid bias.

### Course of the study

The study protocol mandated two primary visits. The initial visit (V1) was scheduled for a mean of 12 weeks (1–68, SD ± 7.55) following infection. The second follow-up visit (V2) was scheduled based on clinical necessity, within a mean interval of 21 weeks (3–50, SD ± 7.96) between the two visits. In a limited number of subjects, additional check-ups were necessary; however, the scope of these additional examinations was limited based on clinical requirements. Both V1 and V2 consisted of an extensive patient interview, documented in free-form by one of the investigating pneumologists (*N* = 14). The focus of these interviews was on the symptoms of acute COVID-19, persisting or newly emerging symptoms, comorbidities, medications, occupational history, and other details customary for a comprehensive pneumological examination. The results of a cardio-respiratory focused physical examination were recorded in free-form according to the standards of the department. The chest X-ray (CXR) in posteroanterior projection was obtained and interpreted by a trained radiologist (*N* = 25). Chest HRCT was performed in selected individuals based on clinical necessity. Pulmonary function testing (PFT) - including spirometry, body-plethysmography, and lung diffusion capacity for carbon monoxide (DLCO) - was conducted by experienced spirometry technicians using the MasterScreen by Jaeger®, with accordance to the recent American Thoracic Society (ATS) and European Respiratory Society (ERS) guidelines [32]. Data retrieval was accomplished through the SentrySuiteTM Version 2.19 by CareFusion. Additionally, a set of blood tests was taken at V1 and V2, including routine biochemistry, differential blood count and SARS-CoV-2 antibodies. However, from October 2021 onwards, the taking of blood tests was limited to cases with clinical necessity due to limited financial resources. The blood tests were therefore not included in statistical analysis to avoid selection bias.

The treatment with glucocorticoids (CS) was considered in patients with persistent pathological findings on CXR and/or HRCT and/or impaired pulmonary function parameters, namely decreased DLCO. The presence of symptoms alone was not considered to be an indication for glucocorticoid therapy. In patients considered for glucocorticoid therapy, additional criteria such as age, frailty, comorbidities, and other individual risk factors were evaluated by the examining physician. Finally, selected patients were prescribed oral glucocorticoids (prednisolone or an equivalent dose of methylprednisolone).

The initial dose and the tapering regimen were individualized, with doses lower than 0.5 mg/kg of prednisolone being utilized in patients with higher estimated risk of adverse effects. The maximum initial dose of prednisolone was 40 mg taken in a single daily dose. The mean prescribed initial dose of prednisolone was 27.6 mg (SD ± 10,64), and the mean duration of glucocorticoid therapy was 13.3 weeks (SD ± 10,06). The dose was reduced to 20 mg after 14 days. The dose of 20 mg was taken for up to 14 days and was then tapered gradually by 5 mg per week. The dose tapering regimen could be modified by the investigating physician. This approach was in line with the national positional document on the treatment of pulmonary impairment in patients recovering from COVID-19 [12].

### Construction and analysis of the dataset

Four main data sources were utilized: the medical documentation, the database of laboratory test results, the database of radiological studies and the Czech national registry of SARS-CoV-2 vaccination and tests. The data were extracted, encoded, and stored in a Microsoft Excel spreadsheet and validated by the main author prior to statistical analysis.

The medical documentation was searched manually, including the reports from the two main visits (V1 and V2) and all interim check-ups. Biometric data were gathered (sex, age, weight, height, BMI). The date of the first positive SARS-CoV-2 test (or onset of symptoms) was extracted from the medical documentation and validated against the Czech national registry of SARS-CoV-2 vaccination and tests. The reasons for missing follow-up were recorded. Selected comorbidities in the patient’s medical history were recorded, including arterial hypertension (AHT), diabetes mellitus (DM), ischemic heart disease (IHD), pulmonary embolism (PE), dyslipidemia (DLD), hypothyroidism (HT), bronchial asthma (BA), chronic obstructive pulmonary disease (COPD), sarcoidosis, interstitial lung disease (ILD). The comorbidity score (ComS) was established by adding one point for each of the ten selected comorbidities.

The severity of acute COVID-19 was classified into one of three categories: mild, defined as a mild ambulatory course without pneumonia; moderate, involving pneumonia but not requiring oxygen therapy; and severe, involving pneumonia that required oxygen therapy, ventilatory support, intensive care, or critical care. The data regarding respiratory and extrapulmonary symptoms during acute COVID-19 and during post-COVID phase were recorded. The rate of subjective improvement was estimated based on the verbal descriptions in the medical documentation and recorded on a scale ranging from 0 to 10 (0 to 100% improvement with 10% step size). SIS scores 9–10 were considered complete improvement, SIS scores 5–8 were considered good improvement, while SIS score of 0 was considered as no improvement. Post-covid radiological score (PRS) was established based on the extent of post-COVID changes as described by the radiologist. The score ranged from 0 (no changes), through 1 (changes on HRCT only), 2 (minimum reticular or linear opacities), 3 (several ground glass opacities, patchy or reticular opacities), 4 (extensive opacities or diffuse reticular opacities in less than 3/6 lung fields) to 5 (changes described in 4 but involving more than 3/6 lung fields). The rate of radiological improvement between the two main visits was evaluated by side-to-side comparison of the radiological studies by a single pneumologist experienced in reading radiological studies and recorded as a scale ranging from 0 to 10 (0 to 100% improvement with 10% step size). RIS scores 9–10 were considered complete improvement, RIS scores 5–8 were considered good improvement, while RIS score of 0 was considered as no improvement. The PFT data encompassed predicted values, actual measured values, and relative values (expressed as a percentage of predicted) where applicable. The data regarding the use of CS were extracted by a pneumologist blinded to the subjective and radiological improvement scores, PFT and laboratory test results. The data included the delay from infection to initiation of therapy, the glucocorticoid molecule used, initial dosage expressed as an equivalent dose of prednisolone, and the duration of the administration in weeks.

The data regarding biometric parameters, subjective symptoms and their improvement, radiological findings, and their improvement, selected PFT parameters and their dynamics, the severity of COVID-19, and the data describing the indication, initial dosage and duration of CS therapy were statistically described and hypotheses were tested by means of univariate analysis. Parametric and non-parametric tests were used as applicable (Student’s t-test, Mann-Whitney U test, Chi-square test). All tests were conducted at the alpha level of 0.05. Logistic regression was employed to investigate potential predictors of a lack of subjective and radiological improvement, defined as a SIS of 0 and a RIS of 0, respectively. The predictors evaluated in the logistic regression included age, sex, COVID-19 severity, the use of glucocorticoids, comorbidity score, and obesity (defined as BMI > = 30). All statistical tests were performed by an experienced statistician using the IBM SPSS Statistics version 29 (IBM Corp., Armonk, NY, USA).

## Results

From May 13, 2020, to January 11, 2022, a total of 2729 survivors of COVID-19 were recruited to the prospective observational study (1247 male, 45.7%). After applying the post-hoc exclusion criteria, 2026 patients (919 male, 45,36%) were included in the statistical analysis, with 131 patients (6.47%) in the CS group and 1895 patients in the WW group. The mean age in the cohort was 54.37 years (SD ± 14.67), with the patients in the CS group being significantly older (mean age 63.44 ± 11.42) than the patients in the WW group (mean age 53.74 ± 14.66; *p* < 0.0001). The BMI was comparable between the study groups (mean 29.52, SD ± 5.87 in the total sample). The patients in the CS group had significantly higher number of comorbidities than the patients in the WW group with the mean comorbidity scores of 1.63 (SD ± 1.25) and 1.02 (SD ± 1.13), respectively (*p* = 0.0004). The patients in the CS group were significantly more likely to be the survivors of severe COVID-19 than the patients in the WW group (70.99% vs. 22.16%, respectively; *p* < 0.0001). The patients in the CS group were significantly more likely to report dyspnea than the patients in the WW group (80.15% vs. 50.18%, respectively; *p* < 0.0001), while the incidence of persistent cough was comparable between the study groups (36.64% vs. 29.97%, respectively; *p* = 0.1384). The values of static lung volumes and parameters of pulmonary diffusion capacity observed at the baseline (V1) were statistically significantly lower in the CS group compared to the WW group (*p* < 0.0001). The post-COVID radiological score was significantly higher in the CS group compared to the WW group with a PRS of 3.75 (SD ± 1.43) and 0.82 ± 1.37, respectively (*p* < 0.0001). See Table 1 for the epidemiological and baseline characteristics of the sample and the subgroups.

**Table 1:** Epidemiological and baseline characteristics of the sample

	total sample (***N*** = 2026)	CS group (***N*** = 131)	WW group (*N*=1895)	***p***-value
	(mean ± SD)	(mean ± SD)	(mean ± SD)	
**Age**	54.37 ± 14.67	63.44 ± 11.42	53.74 ± 14.66	<0.0001^a^
**BMI**	29.52 ± 5.87	29.82 ± 5.37	29.49 ± 5.90	0.5407^a^
**ComS** ^**b**^	1.06 ± 1.15	1.63 ± 1.25	1.02 ± 1.13	0.0004^c^
**PRS** ^**d**^	1.01 ± 1.55	3.75 ± 1.43	0.82 ± 1.37	<0.0001^c^
**VC [%predicted]**	100.52 ± 16.30	86.93 ± 17.25	101.48 ± 15.80	<0.0001^c^
**FEV1/VC [%]**	79.61 ± 6.80	79.96 ± 7.75	79.58 ± 6.73	0.2795^c^
**TLC [%predicted]**	105.37 ± 15.70	90.78 ± 16.36	106.40 ± 15.14	<0.0001^c^
**RV [%predicted]**	126.04 ± 30.42	108.24 ± 28.88	127.29 ± 30.14	<0.0001^c^
**DLCOc [%predicted]**	79.62 ± 16.53	57.69 ± 15.94	81.13 ± 15.46	<0.0001^c^
**KCOc [%predicted]**	89.00 ± 14.78	79.31 ± 16.00	89.67 ± 14.46	<0.0001^c^
	**n (%)**	**n (%)**	**n (%)**	***p*** **-value** ^**e**^
**Male sex**	919 (45.36%)	85 (64.89%)	834 (44.01%)	<0.0001
**Acute COVID-19 severity** ^**f**^				
**mild**	1046 (51.63%)	7 (5.34%)	1039 (54.83%)	<0.0001
**moderate**	433 (21.37%)	27 (20.61%)	406 (21.42%)	0.9812
**severe**	513 (25.32%)	93 (70.99%)	420 (22.16%)	<0.0001
**Persistent respiratory symptoms**				
**dyspnea**	1056 (52.12%)	105 (80.15%)	951 (50.18%)	<0.0001
**cough**	616 (30.40%)	48 (36.64%)	568 (29.97%)	0.1384

^a^Student’s t-test for two samples; ^b^Comorbidity score: calculated by adding 1 point for each of the following selected comorbidities: arterial hypertension, diabetes mellitus, ischemic heart disease, pulmonary embolism, dyslipidemia, hypothyroidism, bronchial asthma, chronic obstructive pulmonary disease, sarcoidosis, interstitial lung disease. The score was available in 652 subjects (46 in the CS group and 606 in the WW group); ^c^Mann-Whitney U test; ^e^Chi-square test; ^f^mild: no pneumonia, no oxygen therapy, moderate: pneumonia not requiring oxygen therapy, severe: pneumonia requiring oxygen therapy

The follow-up data were available in 131 patients in the CS group and 894 patients in the WW group. The pulmonary function test (PFT) results at baseline (V1) and follow-up (V2) visits are presented in the Table 2. Vital capacity (VC) has improved significantly in both the CS group (mean increase by 403.7 ml, SD ± 510.05, or 10.29% of predicted value, SD ± 13.33, *p* = 0.0003 and < 0.0001, respectively) and the WW group (mean increase by 95.1 ml, SD ± 353.23, or 2.68% of predicted value, SD ± 9.94, *p* = 0.0336 and 0.0006, respectively). FEV1/VC (the Tiffeneau index) has significantly decreased in the WW group (-0.73%, SD ± 4.71; *p* = 0.0212), but no significant change was observed in the CS group. Total lung capacity (TLC) has improved significantly in the CS group (mean increase by 649.84 ml, SD ± 756.45, or 9.95% of predicted value, SD ± 12.16, *p* = 0.0001 and < 0.0001, respectively), but not in the WW group (mean increase by 92.72 ml, SD ± 758.67, or 1.27% of predicted value, SD ± 12.84, *p* = 0.2036 and 0.1159, respectively). Residual volume (RV) has increased significantly in the CS group (a mean increase by 204.69 ml, SD ± 700.12, or 8.37% of predicted value, SD ± 29.19, *p* = 0.0366 and 0.0299, respectively). No significant change of RV was observed in the WW group. Significant increase of the transfer factor for carbon monoxide corrected to hemoglobin level (DLCOc) was observed in both the CS group (a mean increase by 10.18% of predicted value, SD ± 12.55, *p* < 0.0001) and the WW group (a mean increase by 2.79% of predicted value, SD ± 12.82, *p* < 0.0101). Neither group has shown a statistically significant increase of the transfer coefficient for carbon monoxide corrected to hemoglobin level (KCOc), with a mean increase by 4.43% of predicted value (SD ± 11.85; *p* = 0.0969) in the CS group, and 1.51% of predicted value (SD ± 10.01; p = 0.5088) in the WW group. Table 3 compares both study groups with respect to the rate of change of the PFT results between the two main visits. The increase of static lung volumes (VC, TLC, RV) was significantly higher in the CS group compared to the WW group (*p* < 0.0001 for VC and TLC, *p* = 0.0013 and 0.003 for absolute and %predicted values of RV, respectively). Similarly, the increase of DLCOc was significantly higher in the CS group (*p* < 0.0001). The differences between the rates of change of FEV1/VC and KCOc were not statistically significant (*p* = 0.1594 and 0.0564, respectively).

**Table 2:** Comparison of pulmonary function test results between the two main visits

		Baseline visit (V1)	Follow-up visit (V2)	Mean difference	***p***-value^**a**^
		(mean ± SD)	(mean ± SD)	MD ± SD	
**CS group (** ***N*** ** = 131)**	**VC [L, ml]**	3.25 ± 0.86	3.65 ± 0.89	403.7 ± 510.05	0,0003
	**VC [%predicted]**	86.93 ± 17.25	97.22 ± 14.96	10.29 ± 13.33	<0.0001
	**FEV1/VC [%]**	79.96 ± 7.75	79.04 ± 7.69	-0.92 ± 5.23	0,3299
	**TLC [L, ml]**	5.68 ± 1.21	6.30 ± 1.25	649.84 ± 756.45	0,0001
	**TLC [%predicted]**	90.78 ± 16.36	100.09 ± 14.86	9.95 ± 12.16	<0.0001
	**RV [L, ml]**	2.48 ± 0.72	2.67 ± 0.71	204.69 ± 700.12	0,0366
	**RV [%predicted]**	108.24 ± 28.88	115.32 ± 27.35	8.37 ± 29.19	0,0299
	**DLCOc [%predicted]**	57.69 ± 15.94	66.90 ± 14.93	10.18 ± 12.55	<0.0001
	**KCOc [%predicted]**	79.31 ± 16.00	83.13 ± 16.51	4.43 ± 11.85	0,0969
**WW group (** ***N*** ** = 894** ^**b**^ **)**	**VC [L, ml]**	3.56 ± 0.98	3.65 ± 0.99	95.1 ± 353.23	0,0336
	**VC [%predicted]**	99.23 ± 16.18	101.91 ± 15.65	2.68 ± 9.94	0,0006
	**FEV1/VC [%]**	79.29 ± 7.00	78.56 ± 6.84	-0.73 ± 4.71	0,0212
	**TLC [L, ml]**	6.11 ± 1.30	6.20 ± 1.34	92.72 ± 758.67	0,2036
	**TLC [%predicted]**	105.16 ± 15.72	106.31 ± 14.65	1.27 ± 12.84	0,1159
	**RV [L, ml]**	2.61 ± 0.71	2.63 ± 0.71	22.18 ± 611.47	0,6972
	**RV [%predicted]**	126.57 ± 30.38	126.59 ± 28.51	0.18 ± 29.61	0,8244
	**DLCOc [%predicted]**	77.79 ± 15.96	80.11 ± 14.71	2.79 ± 12.82	0,0101
	**KCOc [%predicted]**	88.48 ± 15.43	89.10 ± 15.14	1.51 ± 10.01	0,5088

^a^Mann-Whitney U test; ^b^Patients with available follow-up data

**Table 3:** Comparison of CS and WW groups with respect to the changes of pulmonary function

	CS group (***N*** = 131)	WW group (***N***=894^**a**^)	Mean difference	***p***-value^**b**^
	(mean ± SD)	(mean ± SD)	(MD ± SD)	
**ΔVC [ml]**	403.7 ± 510.05	95.1 ± 353.23	308.59 ± 46.10	<0.0001
**ΔVC [%predicted]**	10.3 ± 13.33	2.7 ± 9.94	7.62 ± 1.21	<0.0001
**ΔFEV1/VC [%]**	-0.9 ± 5.23	-0.7 ± 4.71	-0.19 ± 0.48	0,1594
**ΔTLC [ml]**	649.8 ± 756.45	92.7 ± 758.67	557.13 ± 70.79	<0.0001
**ΔTLC [%predicted]**	9.9 ± 12.16	1.3 ± 12.84	8.68 ± 1.15	<0.0001
**ΔRV [ml]**	204.7 ± 700.12	22.2 ± 611.47	182.51 ± 64.50	0,0013
**ΔRV [%predicted]**	8.4 ± 29.19	0.2 ± 29.61	8.19 ± 2.74	0,003
**ΔDLCOc [%predicted)**	10.2 ± 12.55	2.8 ± 12.82	7.39 ± 1.18	<0.0001
**ΔKCOc [%predicted]**	4.4 ± 11.85	1.5 ± 10.01	2.92 ± 1.09	0,0564

^a^Patients with available follow-up data; ^b^Mann-Whitney U test

At the follow-up visit (V2), the mean Radiological Improvement Score (RIS) was 5.9 (SD ± 3.11) and 5.7 (SD ± 3.67) for the CS and WW group, respectively (*p* = 0.7966). Complete radiological improvement (defined as RIS 9 or 10) was observed more frequently in the CS group than the WW group with 21 (16.03%) vs. 94 (10.51%), respectively, but the difference was borderline non-significant (OR 1.62, 95%CI 0.97–2.71). Good radiological improvement (defined as RIS 5 to 8) was observed significantly more frequently in the CS group than the WW group with 64 (48.85%) vs. 98 (10.96%), respectively (OR 7.76, 95%CI 5.19–11.59). The frequency of no radiological improvement (defined as RIS of 0) was comparable between the study groups with 12 (9.16%) cases in the CS group and 55 (6.15%) cases in the WW group, respectively (OR 1.54, 95%CI 0.80–2.96).

Finally, the mean Subjective Improvement Score (SIS) at the follow-up visit (V2) was 7.1 (SD ± 2.71) and 6.7 (SD ± 3.27) for the CS and WW group, respectively (*p* = 0.7). Complete subjective improvement (defined as SIS 9 or 10) was observed significantly more frequently in the CS group than the WW group with 45 (34.35%) vs. 189 (21.14%), respectively (OR 1.95, 95%CI 1.32–2.90). Good subjective improvement (defined as SIS 5 to 8) was seen significantly more frequently in the CS group than the WW group with 66 (50.38%) vs. 196 (21.92%), respectively (OR 3.62, 95%CI 2.48–5.27). The frequency of the subjects reporting no subjective improvement (defined as SIS of 0) was comparable between the study groups with 8 (6.11%) cases in the CS group and 57 (6.38%) cases in the WW group, respectively (OR 0.96, 95%CI 0.44–2.05) (Table 4).

**Table 4:** Comparison of CS and WW groups with respect to the radiological and subjective improvement

	CS group (***N*** = 131)	WW group (***N*** = 894^**a**^)	Mean difference	***p***-value^**c**^
	(mean ± SD)	(mean ± SD)	(MD ± SD)	
**RIS** ^**b**^	5.9 ± 3.11	5.7 ± 3.67	0.17 ± 0.30	0.7966
**SIS** ^**d**^	7.1 ± 2.71	6.7 ± 3.27	0.40 ± 0.26	0.7
	**n (%N)**	**n (%N)**	**OR (95% CI)**	
**Complete radiological improvement:** ^**e**^	21 (16.03%)	94 (10.51%)	1.62 (0.97 - 2.71)	
**Good radiological improvement:** ^**f**^	64 (48.85%)	98 (10.96%)	7.76 (5.19 - 11.59)	
**No radiological improvement:** ^**g**^	12 (9.16%)	55 (6.15%)	1.54 (0.80 - 2.96)	
**Complete subjective improvement:** ^**h**^	45 (34.35%)	189 (21.14%)	1.95 (1.32 - 2.90)	
**Good subjective improvement:** ^**i**^	66 (50.38%)	196 (21.92%)	3.62 (2.48 - 5.27)	
**No subjective improvement:** ^**j**^	8 (6.11%)	57 (6.38%)	0.96 (0.44 - 2.05)	

^a^Patients with available follow-up data; ^b^Radiological improvement score (0–10); ^c^Mann-Whitney U test; ^d^Subjective improvement score (0–10); ^e^RIS 9–10; ^f^RIS 5–8; ^g^RIS = 0; ^h^SIS 9–10; ^i^SIS 5–8; ^i^SIS = 0

Logistic regression analysis was conducted to evaluate the likely predictors of lack of subjective and radiological improvement, defined as SIS = 0 and RIS = 0, respectively. Age, sex, COVID-19 severity, the use of CS, Comorbidity score (ComS), and obesity were selected as independent variables. The only independent variable that significantly predicted the lack of subjective improvement was the Comorbidity score, where a unit increase in the score increased the odds of the patient reporting no subjective improvement 1.307 times (95% CI 1.031–1.658; *p* = 0.027). The only independent variable that significantly predicted the lack of radiological improvement was the increasing severity of acute COVID-19, with OR 0.271 (95% CI 0.119–0.618) and 0.323 (95% CI 0.156–0.671) for a moderate and severe course of COVID-19, respectively (*p* = 0.002).

## Discussion

Both the patients receiving oral CS and the patients in the watchful waiting group have seen statistically significant increases in static lung volumes and DLCO. Most of the baseline characteristics (Age, BMI, VC, FEV1/VC, DLCO, KCO) in our study were very close to the characteristics of the smaller cohort (*n* = 35) described by Myall et al. [5] offering a good opportunity for comparison. The rate of improvement of vital capacity (9.6% of predicted value, SD ± 13.6) reported in the aforementioned study was comparable to our results, however, the reported improvement of DLCO (31.49% of predicted value, SD ± 27.7) was much higher than in our study, albeit with higher variance probably due to smaller sample size. Interestingly, the study reported a significant improvement in KCO by a mean of 19.9% of predicted (95% CI 9.72–30.1), with no significant improvement being observed in our cohort. The authors briefly state that the functional improvement in their cohort was mirrored by good resolution of radiological changes and clinical improvement, which seems to be in line with our cohort, where complete and good radiological and subjective improvement was frequently observed in the CS group. Myall et al. suggest CS therapy only for symptomatic patients with involvement of more than 15% of lung parenchyma on chest CT, with the lack of spontaneous improvement. The initial dosage was similar to our study (a mean dose of 26.6 vs. 27.6 mg of prednisolone, respectively). We speculate that one of the reasons for better improvement of DLCO and KCO reported by Myal et al. may be the earlier initiation of therapy (6 weeks vs. a mean of 12 weeks, SD 7.55), and perhaps even the length of therapy (3 weeks vs. a mean of 21 weeks, SD ± 7.96). Furthermore, the more restrictive indication of VC therapy based on 6-minute walk test (6MWT), or the presence of desaturation > = 4% may have been a more precise selection tool for the consideration of CS therapy, indicating possible overprescription in our cohort, where the criteria were less strict (decreased DLCOc, persistent radiological abnormalities and persistent respiratory symptoms). We acknowledge the lack of 6MWT as one of the weaknesses of our study. Importantly, our study shows that patients in the CS medicating group displayed significantly better rates of improvement of static lung volumes and DLCO compared to the watchful waiting group, and that the difference seems to be large enough to be clinically relevant.

Dhooria et al. [31] conducted an investigator-initiated, single-center, open-label, parallel-group, randomized, superiority trial (the COLDSTER trial) comparing two prednisolone dosage regimens in 130 patients with post-COVID diffuse lung abnormalities (PC-DPLAS). The CS therapy was initiated 3–8 weeks post-COVID - earlier than in our study. The subjects were randomized into two equally sized groups to receive either the high-dose regimen (initial dose of 40 mg of prednisolone tapered over 6 weeks) or the low-dose regimen (10 mg of prednisolone over 6 weeks). The inclusion criteria were diffuse lung abnormalities affecting at least 20% of the lung parenchyma on semiquantitative assessment on thin section CT, and at least one of the following: mMRC score of 2 or higher, resting SpO2 94% or below, or desaturation by at least 4% during 6MWT. No significant difference was observed between the two CS regimens with regards to the rate of complete radiological response (defined as 90% or greater improvement on the follow-up chest CT scan), which was seen in 24.6% and 18.5% in high and low-dose CS groups, respectively. Similarly, no significant difference between the study groups was observed with respect to the rate of “good or complete radiological response” (defined as 50% or greater improvement), which was seen in 84.6% and 80.0% in the high and low dose regimen, respectively. The reported rates of radiological response were higher than in our cohort, where the complete response in the CS group was observed in 16.03% and “complete or good” response in 48.85%. However, the comparison is not direct as the radiological improvement in our cohort was scored semi-quantitatively using paired chest X-rays rather than the quantitative assessment of the CT scans used in the COLDSTER trial. Despite this major methodological difference, the results consistently show that the majority of patients prescribed CS have experienced significant radiological improvement. Importantly, our data show that the rates of “good or complete” radiological improvement were significantly higher in the CS versus watchful waiting group. Unfortunately, the PFT in the COLDSTER trial only encompassed standard spirometry and only the value of FVC at week 6 was reported, without providing the baseline value. The reported mean FVC at week 6 is significantly lower even in comparison with the mean baseline value observed in our cohort, even more so when compared to the value at V2. Additionally, there were significant differences between the study population and our cohort with respect to sex, age and COVID-19 severity.

Goel et al. [30] reported on 49 patients with long COVID, abnormal findings on chest CT, and resting or exertion hypoxemia. 24 of the patients were treated with deflazacort for 8 to 10 weeks, initiated 4 weeks after the infection - earlier than in our cohort. At the initiation of therapy, 58% of the patients reported Modified Medical Research Council (mMRC) score of 4, which decreased to a score 2 or less in 86% of the patients following CS treatment. The occurrence of breathlessness has halved, the incidence of cough has decreased by almost 70%. The majority of the patients (71%) reported subjective improvement, which is in line with patients in our cohort, where 50.38% and 34.35% of patients treated with CS have reported good and complete subjective improvement, respectively. Complete radiological resolution of post-COVID residual changes was seen in 25% of the patients, which is closer to the 16.03% of patients with complete radiological response in the CS group of our cohort. Additionally, Goel et al. report the 6-minute walk test distance (6MWD) increase by an average of 75 m. Indication criteria for CS therapy were abnormalities on HRCT of the lungs (reticulations, ground glass opacities or parenchymal bands) and oxygen saturation (SpO2) below 90%, or desaturation by more than 4% during 6MWT. Deflazacort was administered in a dose equivalent to 0,25–0,5 mg/kg of prednisolone, which is comparable to our study, and tapered over 6 to 10 weeks (usually 8 weeks) based on radiological recovery. The weakness of the study was the lack of pulmonary function testing and the small cohort size.

According to Bieksiene et al. [20] timely glucocorticoid therapy can be beneficial in preventing the development of definitive fibrotic changes in patients with post-COVID pulmonary impairment, typically within the first 12 weeks from the acute stage of the disease. In the case of more dramatic late-stage disease presentations, such as acute fibrinous organizing pneumonia, several cases of good response to corticosteroid pulses (up to 1 g methylprednisolone for 3 consecutive days) followed by tapering to lower doses of CS have been reported in the literature [23, 24, 33].

### Limitations of the study

One of the major limitations of the present study is the fact that the data extraction process is still ongoing. The results of the study should therefore be viewed as interim. This is partially outweighed by the relatively large size and robustness of the available data. Another weakness of this study is the lack of randomization as this was a real-life study and the decision for CS therapy was driven by perceived clinical necessity with a primary intention not to harm. The dosage of CS used in the study was not standardized and was subject to the decision of the investigating physician. Finally, the currently incomplete data regarding the course of acute COVID-19 only permitted a rough division into mild, moderate and severe categories, but did not provide enough data to accurately label the survivors of critical COVID-19. This will likely change in the future as more data are being extracted from the source information.

## Conclusion

Despite the marked differences between study populations and methodological differences, most of the published studies report favorable radiological, subjective, and functional outcomes in most patients treated with glucocorticoids for post-COVID pulmonary involvement. Our study confirms these previous observations by providing a comparatively large dataset and a comparison to an unmatched control group, showing better functional, radiological, and subjective improvement in selected patients treated with oral corticosteroids.

Considering our data alongside previously published studies, the suggested indication criteria should include significant parenchymal involvement, decreased value of DLCO and persistent subjective symptoms. Resting desaturation, or desaturation during the 6-minute walk test may further support the decision and lead to more precise indication of CS therapy.

One possible approach to improve clinical decision making may be the use of artificial intelligence driven algorithms, as demonstrated by an affiliated collective of Myska et al. [34], who have used the data from Olomouc post-COVID dataset to test the performance of 9 different machine learning algorithms in predicting improvement in the patients indicated for CS therapy. The best results were achieved by the Decision Tree approach, reaching 73.52% balanced accuracy on a validation portion of the dataset.

The authors continue with the works on the Olomouc post-COVID dataset, aiming at providing more detailed data on comorbidities and other clinical parameters. The authors aim to make the dataset publicly available and to provide data from further follow-up to hopefully bring new insights about the more long-term outcomes of these patients, as there are still questions to be answered. The topics for future research include, among others, the optimum length of therapy, the criteria for cessation of therapy, the possible benefits of antifibrotics in combination with CS, or the incidence and characteristics of relapses following CS cessation.

## Data Availability

The data that support the findings of this study are not openly available due to reasons of sensitivity and are available from the corresponding author upon reasonable request. Data are located in controlled access data storage at Palacky University Olomouc, Czech republic.
